# Severe hypoglycemia with reduced liver volume as an indicator of end-stage malnutrition in patients with anorexia nervosa: a retrospective observational study

**DOI:** 10.1186/s40337-024-01011-1

**Published:** 2024-05-03

**Authors:** Hidenori Matsunaga, Keisen Riku, Kentaro Shimizu, Satoshi Fujimi

**Affiliations:** 1 Department of Psychiatry, Osaka General Medical Center, Bandai-Higashi 3-1-56, Sumiyoshi-ku, Osaka, 558-8558 Japan; 2 Department of Psychiatry, Osaka University Graduate School of Medicine, Yamada-Oka 2-2, Suita-City, Osaka 565-0871 Japan; 3 Department of Pharmacotherapeutics II, Faculty of Pharmacy, Osaka Medical and Pharmaceutical University, Nasahara 4-20-1, Takatsuki-City, Osaka 569-1094 Japan; 4 Rikusato Kenko Clinic, Andoji-Machi, 2-6-3-102, Chuo-ku, Osaka, 542-0061 Japan; 5 Department of Traumatology and Acute Critical Medicine, Osaka University Graduate School of Medicine, Yamada-Oka 2-2, Suita-City, Osaka 565-0871 Japan; 6 Division of Trauma and Surgical Critical Care, Osaka General Medical Center, Bandai-Higashi 3-1-56, Sumiyoshi-ku, Osaka, 558-8558 Japan

**Keywords:** Anorexia nervosa, Severe malnutrition, Hypoglycemia, Low serum triglycerides, Liver failure, Liver volume, Liver autophagy, Refeeding syndrome

## Abstract

**Background:**

Hypophosphatemia due to excessive carbohydrate administration is considered the primary pathogenesis of refeeding syndrome. However, its association with liver injury and hypoglycemia, often seen in severe malnutrition before re-nutrition, remains unclear. Autophagy reportedly occurs in the liver of patients with severe malnutrition. This study aimed to clarify the pathophysiology of liver injury and hypoglycemia by focusing on liver volume.

**Methods:**

Forty-eight patients with anorexia nervosa with a body mass index (BMI) of < 13 kg/m^2^ were included (median BMI: 10.51 kg/m^2^ on admission). Liver volume was measured in 36 patients who underwent abdominal computed tomography (CT), and the “estimated liver weight/ideal body weight” was used as the liver volume index. Seventeen blood test items were analyzed during the first 60 days.

**Results:**

Liver volume significantly decreased when abdominal CTs were conducted shortly before or after hypoglycemia compared to when the scans were performed during periods without hypoglycemia. Five patients with severe hypoglycemia on days 13–18 after admission had a very low nutritional intake; of them, four showed a marked decrease in liver volume. Severe hypoglycemia was accompanied by low serum triglycerides and liver dysfunction. Patients experiencing hypoglycemia of blood glucose levels < 55 mg/dL (< 3.05 mmol/L) (32 patients; median lowest BMI: 9.45 kg/m^2^) exhibited significantly poorer blood findings for most of the 17 items, except serum phosphorus and potassium, than did those not experiencing hypoglycemia (16 patients; median lowest BMI: 11.2 kg/m^2^). All patients with a poor prognosis belonged to the hypoglycemia group. Empirically, initiating re-nutrition at 500 kcal/day (20–25 kcal/kg/day), increasing to 700–800 kcal/day after a week, and then gradually escalating can reduce serious complications following severe hypoglycemia.

**Conclusions:**

Liver volume reduction accompanied by hypoglycemia, low serum triglyceride levels, and liver dysfunction occurs when the body's stored energy sources are depleted and external nutritional intake is inadequate, suggesting that the liver was consumed as a last resort to obtain energy essential for daily survival. This pathophysiology, distinct from refeeding syndrome, indicates the terminal stage of malnutrition and is a risk factor for complications and poor prognosis. In treatment, extremely low nutrient levels should be avoided.

## Abstract (plain language summary)

This study aimed to clarify the pathophysiology of severe malnutrition in patients with anorexia nervosa by focusing on liver volume. The small size of the liver was almost always accompanied by hypoglycemia within a week. In several cases, extremely low nutritional intake, continued for approximately 2 weeks after admission, resulted in severe hypoglycemia and a marked decrease in liver volume. The 32 patients with hypoglycemia presented worse blood test items related to liver function, nutrition, and blood cell count compared to the 16 patients without such a condition. All cases with poor prognosis were in the hypoglycemia group. These findings suggest that severe hypoglycemia with decreased liver volume indicates the end stage of malnutrition. Liver volume reduction is considered a reflection of the liver's consumption of itself as a last resort for energy procurement for daily survival when the body's stored energy sources are depleted, and external nutritional intake is insufficient. When managing such patients, extremely low nutritional administration should be avoided.

## Background

Refeeding syndrome is a well-known physical pathophysiology in severe malnutrition. It is a potentially fatal complication that can occur during refeeding of patients with severe malnutrition [1–6]. Therefore, measures such as careful nutritional administration, monitoring, and supplementation of phosphorus (P) and other electrolytes are carefully implemented for the management of such patients [2–7]. Moreover, severe liver injury and hypoglycemia are occasionally observed in patients with severe malnutrition [8–14]; however, the underlying mechanisms are not fully understood.

The refeeding syndrome was first described in the 1970s and 1980s with the development of total parenteral nutrition when cases of death from severe circulatory and respiratory failure occurred within 1 or 2 days following the initiation of high-calorie carbohydrate infusions, such as 2000 or 3000 kcal/day, in severely malnourished patients [1]. This syndrome is considered to be induced by an increase in insulin secretion due to excessive ingestion of carbohydrates and the subsequent intracellular transfer of P, magnesium (Mg), and potassium (K), resulting in decreased levels of these electrolytes in the blood, which, along with these metabolic abnormalities, causes damage to various organs. This can subsequently lead to heart failure, edema, muscle weakness, and central nervous system dysfunction [6]. Furthermore, Boateng et al. [6] posit that fatty liver occurs due to excessive carbohydrate intake.

According to the recommendations for refeeding syndrome by the American Society for Parenteral and Enteral Nutrition in 2020 [15], the diagnostic criteria for refeeding syndrome can be stratified as follows: "a decrease in any 1, 2, or 3 of serum phosphorus, potassium, and/or magnesium levels by 10–20% (mild), 20–30% (moderate), or > 30% and/or organ dysfunction resulting from a decrease in any of these and/or due to thiamine deficiency (severe), occurring within 5 days of reintroduction of calories.” Importantly, the primary focus of these criteria is on electrolyte laboratory abnormalities. Specifically, the signs and symptoms enumerated pertain solely to those associated with electrolyte imbalances and do not encompass liver injury and hypoglycemia.

However, liver injury is often observed in malnourished patients with anorexia nervosa (AN), reportedly occurring before refeeding [8–14]. Notably, several studies have shown an association between liver injury and low body mass index (BMI) [8, 9, 12–14], with some of them highlighting that severe elevation in liver enzyme activities is associated with hypoglycemia [11, 13, 14].

In 2008, Rautou et al. [16] reported the autophagy findings of livers of patients with AN and liver failure. Specifically, they performed liver biopsies on 12 patients with AN admitted to the emergency room with a prothrombin index < 50% and found no evidence of necrosis or apoptosis in hepatocytes. Electron microscopy of tissues from four of these patients revealed autophagy in all four cases. Importantly, autophagy is a physiological function through which body tissues are used as an energy source during starvation, and it does not involve cell death [17]. However, hepatocyte death occurs when malnutrition becomes severe [17, 18]. Another potential pathophysiological mechanism of liver injury is hepatic hypoperfusion due to severe dehydration during serious malnutrition [19].

In Japan, there are no specialized medical institutions to treat AN, and the treatment is left to the discretion of each medical institution. The Osaka General Medical Center (OGMC) is one of the few general hospitals in Osaka having both an emergency center and a psychiatric ward, with the latter exclusively accepting psychiatric patients who require inpatient treatment for both their mental and physical conditions. Since 2008, patients with severe AN have been admitted to the hospital annually. The Physical Medicine Department initially takes charge of treating physical complications and re-nutrition, and nutrition is commenced at very low doses, as per the guidelines published by the National Institute for Health and Care Excellence (NICE). However, in some instances, patients died due to complications before nutrition was sufficiently increased. Based on this experience, the Psychiatry Department began to take charge of nutritional management in 2013, considering that increasing the amount of nutrients administered is necessary to overcome the physical crisis. Furthermore, the objective during hospitalization is to elevate the patient's weight to a safer range before discharge rather than releasing patients immediately after the crisis has been managed.

In our experience with severe AN, severe emaciation was strongly associated with liver damage and hypoglycemia, which coincides with the findings of previous studies [11, 13, 14]. In addition, we noted the small size of the liver on abdominal computed tomography (CT) in severely malnourished patients. Because the liver is a glucose-regulating organ itself, supplying glucose to and taking it up from the blood, we considered that severe hypoglycemia occurs due to the failure of this function of the liver as well as the lack of energy sources. Furthermore, although the liver has ample spare capacity, liver volume is proportional to liver function, and a decrease in liver volume directly implies a decrease in organ function. Notably, in liver surgery, liver function, and remnant liver volume are critical factors for preventing post-operative liver failure [20]. Based on these findings, we hypothesized that hypoglycemia and liver failure in severely malnourished patients might be related to the decreased size of the liver.

The primary objective of this study was to investigate hypoglycemia and liver injury, which cannot be explained by refeeding syndrome, from the perspective of liver volume. Furthermore, we aimed to examine the influence of hypoglycemia on the risk of complications that occur during refeeding by extracting the poorest values of blood investigation abnormalities during the first 60 days of treatment.

## Methods

### Participants

**Fig. 1:**
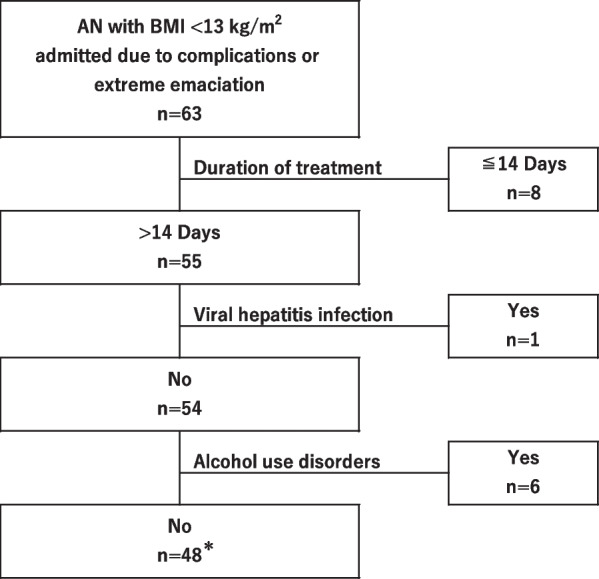
Diagram of patient selection process. *Twenty-five of them were first admitted to other institutions and transferred to OGMC. AN: anorexia nervosa. BMI: body mass index

Among patients with AN admitted to the OGMC between January 1, 2008, and March 31, 2022, 63 were recruited based on the following inclusion criteria: a BMI < 13 kg/m^2^ during the admission course and admission due to serious physical complications or a high risk thereof resulting from extreme emaciation. All of them were treated by the Psychiatry Department, and most of them also by the Physical Medicine Department. Figure 1 shows a flow chart depicting patient recruitment. To investigate the physical complications over a sufficient treatment period, eight patients were excluded because they had been treated for less than 14 days. Additionally, one patient with a history of hepatitis B and C and six patients with alcohol dependence were excluded because the main purpose of the study was to analyze the liver. Finally, a total of 48 patients were included in this study; of them, 25 were transferred from other hospitals.

The study was conducted in accordance with the Declaration of Helsinki and approved by the Institutional Ethics Committee of OGMC (protocol code: 28-S1102; date of approval, November 25, 2016). This study was conducted in 2016 and 2018 for presentation at conferences, utilizing retrospective data from 2008. For the current article, we have gathered cases from 2018 through 2022 and analyzed them collectively. Patient consent was waived due to the retrospective nature of the study.

An age- and sex-matched control group was recruited from psychiatric inpatients admitted to OGMC to compare liver volume. Importantly, these individuals' psychiatric and physical illnesses were not deemed to significantly impact liver volume. Moreover, patients with fatty liver findings were excluded. Notably, physical illnesses in the control group included crash trauma, medication overdose, autoimmune encephalitis, pneumonia, cancer, and others, whereas mental illnesses comprised schizophrenia, mood disorders, and organic brain syndromes.

### Data collection

This retrospective study collected clinical data encompassing age, sex, weight, AN subclass, disease duration, clinical course (including transfusion of blood products), blood test and CT scan results, and prognosis.

Because this study aimed to examine the pathophysiology of severe malnutrition, data on the transferred patients were collected from the previous hospitals and included in the analysis. Notably, in cases of multiple hospitalizations due to separate episodes of exacerbation, those with more complete data were included in the analysis. If a patient was discharged from the hospital and immediately readmitted, it was considered the same episode, and both hospitalizations were included in the analysis. Finally, laboratory data from the outpatient setting just before admission were also included in the analysis, if available.

For the blood investigation results, in order to examine physical complications in the course of treatment, the poorest (highest or lowest) data values within the first 60 days of the episode, as well as the results on the first day, were extracted for 17 blood investigation items; the actual data analysis period was 60 days for 34 patients and 22–59 days for 14 patients. The 17 blood investigation parameters included albumin, prealbumin, triglycerides (TGs), blood glucose (BG), total bilirubin, aspartate aminotransferase (AST), alanine aminotransferase (ALT), cholinesterase (ChE), prothrombin time (PT; %), P, Mg, K, C-reactive protein, white blood cell count, hemoglobin, platelet count, and total lymphocyte count. The patients were divided into two groups, with or without hypoglycemia (BG < 55 mg/dL (3.05 mmol/L)).

Focusing on the nutritional status in relation to hypoglycemia and liver injury, we selected the results of BG, blood TGs, and ALT from the day of admission for 28 patients who had all three results.

The liver volume was assessed using SYNAPSE VINCENT® (Fujifilm Medical Co., Tokyo, Japan) from abdominal CT images; in some countries, it is referred to as SYNAPSE 3D®. Abdominal CT images were stored as 5-mm slices until January 2018 and as 1-mm slices thereafter, and these data were used for measurement. The manufacturer informed us that the measurement error due to differences in slice thickness was within 2%. When the software recognized some other part to be the liver, it was corrected manually. Moreover, the intra- and peri-hepatic portions of the inferior vena cava were manually excluded. All liver volumes were re-measured in an identical manner using the method indicated by the software manufacturer. Because liver size is affected by body size, the “estimated liver weight/ideal body weight” (eLW/IBW; %) was used as an index of liver size. A BMI of 22 kg/m^2^ was regarded as an ideal body weight.

Importantly, Inai et al. [21] reported that the specific gravity of the liver for most conditions and fatty liver was in the range of 1.050–1.060 and 1.020–1.030, respectively. Based on these findings, the specific gravity of the liver was determined to be 1.055. The eLW/IBW formula is as follows: eLW/IBW = liver weight (liver volume × 1.055)/IBW. To avoid complications and to use the index to indicate liver volume, specific gravity was fixed at 1.055 and calculated, even in the presence of fatty liver findings.

The volumes of pleural effusion, ascites, and pyothorax were measured using SYNAPSE VINCENT® and were utilized to adjust the patients’ body weight.

For the five patients who exhibited severe hypoglycemia 13–18 days post-admission, the quantity of nutrition administered from admission until the onset of severe hypoglycemia was recorded.

Owing to the retrospective study design, several parameters in blood investigations, such as TGs, prealbumin, ChE, and total lymphocyte count, were sometimes missing, whereas CT was not performed in nearly half of the cases without hypoglycemia. For five of the 25 patients transferred from other hospitals, the blood test results for days 6, 10, 13, 13, and 20 were unavailable. Moreover, three of these patients showed abnormal results in several parameters during treatment in OGMC, and the other two had not had physical complications in the previous institution and exhibited mostly stable blood test results at our hospital. Therefore, we determined that the available data did not constitute a significant obstacle.

### Statistical analysis

The Mann–Whitney U test was used to analyze the difference in numerical data between the two groups. Spearman’s rank correlation coefficient test was applied to assess the correlations between BG and blood TGs and ALT at admission. Moreover, regression analysis was employed to examine the correlation between liver volume and BMI. Furthermore, the chi-square test was used to determine the difference in frequency between the two groups. SPSS version 27 (IBM Corp., Armonk, NY, USA) was used for all statistical analyses. Two-sided p-values < 0.05 were considered statistically significant.

## Results

### Patients’ characteristics

The baseline characteristics of the total 48 participants and 36 patients with abdominal CT, as well as age, sex, and BMI of control patients for liver volume, are presented in Table 1. Regarding BMI at the time of admission, three patients had severe edema when arriving from other hospitals and were treated as missing values because the amount of excess fluid was unknown. For the four cases with massive pleural and peritoneal effusions, the measured volumes of effusions were 1300 mL, 1700 mL, 4100 mL, and 8300 mL. Additionally, for the one case with a large pyothorax, the measured volume was 3700 mL. These volumes were subtracted from the body weight for each case, assuming the specific gravity to be 1. Other complications associated with hospitalization (Table 1) included bradycardia, renal failure, liver dysfunction, massive ascites, confusion, and mental instability. Notably, based on the BMI at discharge, eight patients with poor prognosis were excluded.

**Table 1:** Baseline characteristics of the study participants

	Total participants	With abdominal CT	Control patients for liver volume
	n = 48	n = 36	n = 50
Sex: male/female	2/46	2/34	2/48
Age	32	34	34.5
Subtypes of anorexia nervosa Restrictive type/(Binge-eating/purging type)	37/11	31/5	–
Duration of the disease (years)	6	6	–
The first unit of admission in OGMC (Emergency/Other physical/Psychiatric)	17/3/28	16/2/18	–
Duration of admission in previous hospitals (days)	14 (n = 25)	11 (n = 18)	–
Duration of admission in OGMC (days)	96	99	-
BMI			
At the time of admission to OGMC	10.51 (n = 45)	10.18 (n = 33)	–
At the time of abdominal CT	–	–	20.85
The smallest during the first 2 months	9.95	9.60	–
At the time of discharge from OGMC	13.35 (n = 40)	13.25 (n = 40)	–
Main reasons for admission			
Hypoglycemia with loss of consciousness	13	10	–
Hypokalemia	5	3	–
Infection	5	5	–
Other complications	16	11	–
Severe malnutrition itself	9	7	–
With hypoglycemia within the first 60 days	32	27	–
Prognoses at the discharge from OGMC [died/hypoglycemic encephalopathy] /well	[6/2]/40	[6/2]/28	–

Hypoglycemia is defined as blood glucose levels < 55 mg/dL (< 3.05 mmol/L). Numerical data are expressed as median

BMI: body mass index, CT: computed tomography, OGMC: Osaka General Medical Center

**Fig. 2:**
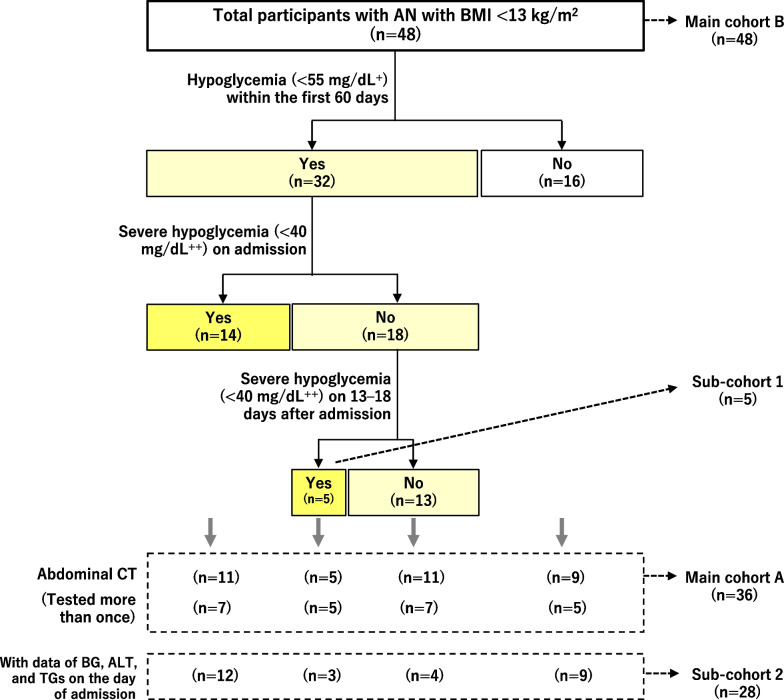
Cohorts analyzed in this study. The yellow color indicates patients with hypoglycemia. ^+^55 mg/dL is equivalent to 3.05 mmol/L. ^++^40 mg/dL is equivalent to 2.22 mmol/L

Figure 2 shows the grouping of all participants in terms of hypoglycemia, as well as cohorts analyzed in this study.

### Liver volume and hypoglycemia

**Fig. 3:**
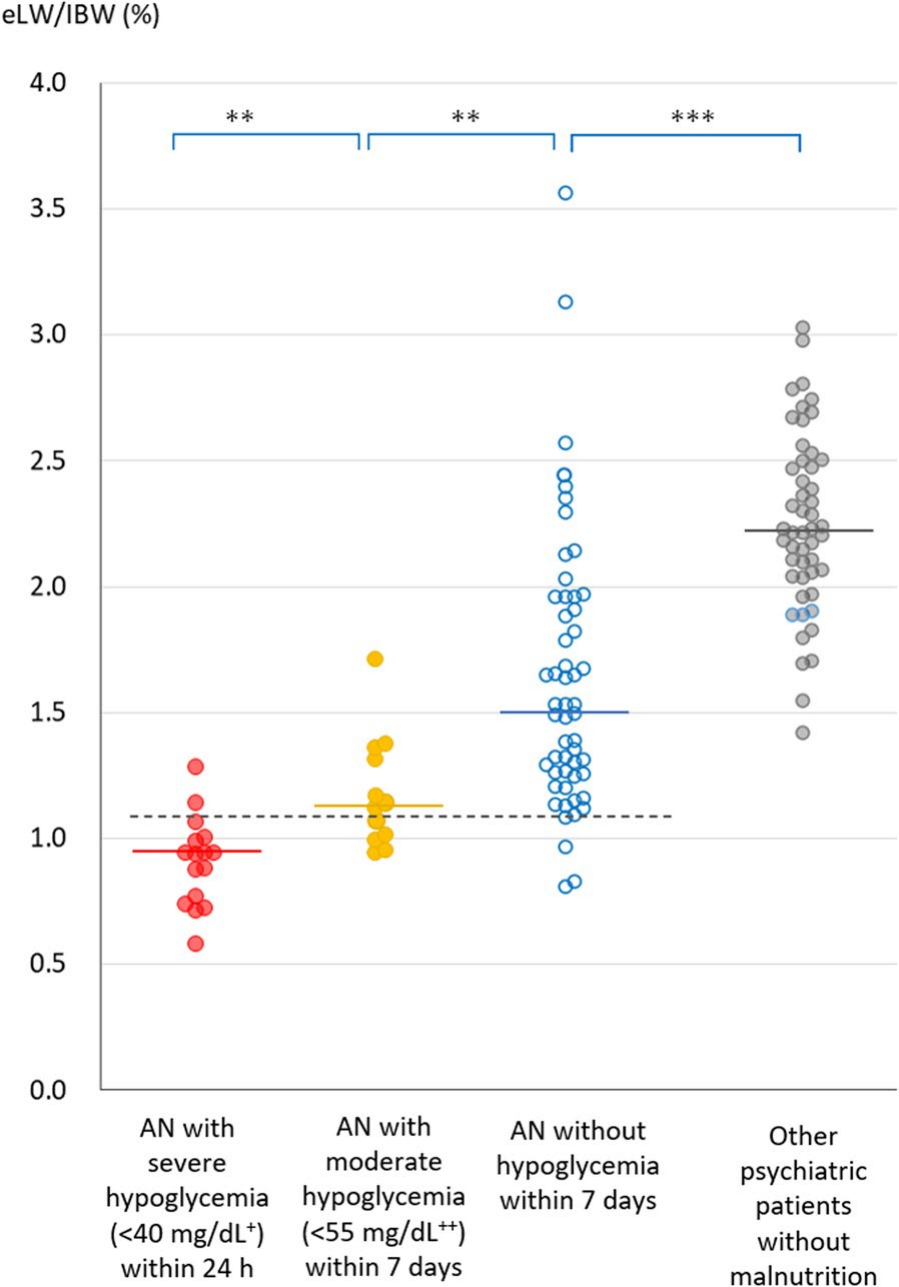
Liver volumes in patients with anorexia nervosa with and without hypoglycemia and in control patients. The medians of the severe, moderate, and without hypoglycemia groups and the control group are 0.94, 1.13, 1.51, and 2.22, respectively. **p < 0.01, ***p < 0.001. The threshold increasing the risk of hypoglycemia is 1.08%. ^+^40 mg/dL is equivalent to 2.22 mmol/L. ^++^55 mg/dL is equivalent to 3.05 mmol/L. eLW/IBW: estimated liver weight/ideal body weight, AN: anorexia nervosa

Abdominal CT was performed in 36 of 48 patients (27 of 32 with hypoglycemia and nine of 16 without hypoglycemia), 24 of whom underwent more than one CT (19 with hypoglycemia and five without hypoglycemia). All data for eLW/IBW, an index of liver size obtained from CT, are shown in Fig. 3, as well as 50 other psychiatric patients without malnutrition for controls. Data of patients with AN were divided by the presence and degree of hypoglycemia.

There was a significant difference in liver size among the three AN groups: between severe and moderate (*p* = 0.002) and between moderate and no hypoglycemia (*p* = 0.002). Notably, a significant difference was also observed between AN without hypoglycemia and the control group (*p* < 0.001). Moreover, eLW/IBW < 1.08% was observed in 14 of 16 patients with severe hypoglycemia and three of 55 patients without hypoglycemia, indicating that an eLW/IBW of 1.08% was the threshold at which a higher rate of severe hypoglycemia occurred.

**Fig. 4:**
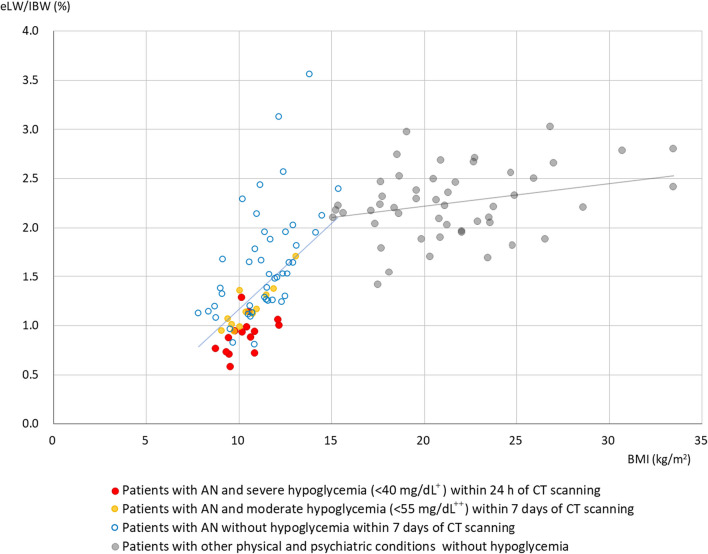
Liver size, body mass index, and hypoglycemia. Seventy-two data points from 35 patients with AN and 50 data points from 50 control participants are plotted. Two lines indicate regression lines of the AN and control groups. The regression line of the AN group was obtained by excluding three data points higher than 2.5% with fatty liver. ^+^40 mg/dL is equivalent to 2.22 mmol/L. ^++^55 mg/dL is equivalent to 3.05 mmol/L. eLW/IBW: estimated liver weight/ideal body weight, BMI: body mass index, AN: anorexia nervosa

The association between liver volume and BMI in patients with AN and controls is illustrated in Fig. 4. Of the 82 data points from 36 patients with AN, 10 data points from six patients whose weight at the time of CT was unknown were excluded. Consequently, 72 data points from 35 patients were utilized; weights of patients with massive pleural and peritoneal effusion, as well as those with large pyothorax, were adjusted by subtracting the weight of extra fluid. Three CT scans from two patients with AN revealed enlarged livers (three CT scans with eLW/IBW > 2.5%); however, both patients had fatty livers. The liver volume of control patients slightly decreased with decreasing BMI (regression coefficient: 0.03, *p* < 0.05); in contrast, that of patients with AN, including those with and without hypoglycemia, showed a marked decrease with decreasing BMI (regression coefficient: 0.17, *p* < 0.001, excluding three data points with fatty liver).

### Five patients who developed severe hypoglycemia after admission

**Fig. 5:**
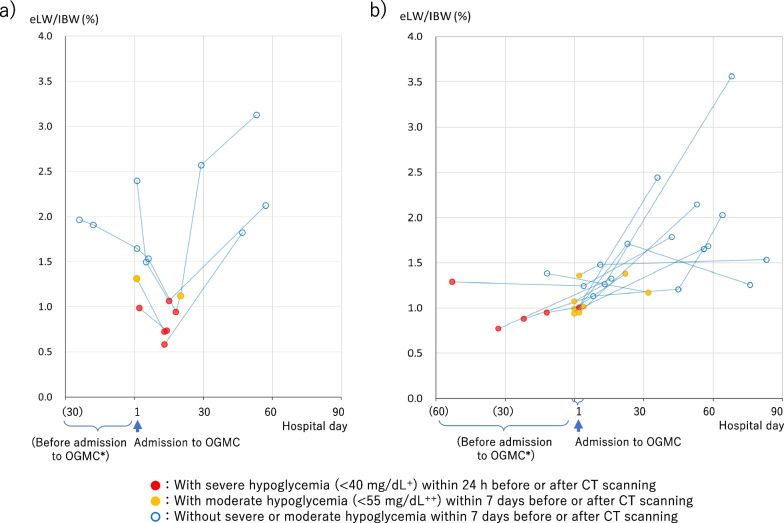
Serial liver volume according to the day of admission to Osaka General Medical Center. **a** Results for five patients exhibiting severe hypoglycemia 13–18 days post-admission to OGMC. **b** Serial outcomes for the other 11 patients with serial data and with hypoglycemia. *Data collected during admission to previous hospitals or in an outpatient setting. Results for the same patients are linked by lines. To prevent overlap, dots representing the day of admission are spaced out over 2 days. ^+^40 mg/dL is equivalent to 2.22 mmol/L. ^++^55 mg/dL is equivalent to 3.05 mmol/L. eLW/IBW: estimated liver weight/ideal body weight, CT: computed tomography, OGMC: Osaka General Medical Center

The serial liver volume data for the five patients who experienced severe hypoglycemia 13–18 days post-admission to OGMC is presented in Fig. 5a. For comparison, Fig. 5b illustrates the data for the remaining 11 patients who experienced hypoglycemia within a week around CT and had serial liver volume data. Notably, four of the five patients demonstrated a significant reduction in liver volume compared to that at the time of admission. Notably, follow-up CT scans after recovery from severe hypoglycemic episodes revealed an increased liver volume in almost all cases, with some showing an enlargement of the liver (Fig. 5a, b).

Details of the five patients who developed severe hypoglycemia 13–18 days after admission to OGMC are shown in Table 2. The amount of nutrition administered was low in all five patients, and in four of them, it was accompanied by factors that increased the metabolic rate. In three of them, the TG levels were also extremely low.

**Table 2:** Details of five patients who showed severe hypoglycemia 13–18 days after admission

No	Day X* (Hospital day)	Blood investigation results			Body weight (BMI)		Average amount of nutrition from Day 2 to Day X		eLW/IBW		Factors increasing energy consumption (highest CRP: mg/dL)
		BG	TGs	ALT	Day 1	Day X	kcal/day	kcal/kg/day**	Day 1	Day X	
		mg/dL	mg/dL	U/L	kg (kg/m^2^)	kg (kg/m^2^)			%	%	
1	13	23	1	156	27.5 (11.45)	26 (10.82)	450	16.8	1.31	0.72	Pneumonia (13.4)
2	16	21	17	1331	35.0 (15.56) ***	27.2 (12.09)	511	16.4	2.4	1.07	Large lung abscess (19.4)
3	14	32	–	19	26.6 (11.99) ***	23.8 (9.30) ***	252	10.0	0.99	0.74	Massive pleural effusion, ascites, and pneumonia (14.4)
4	13	9	–	458	27 (10.40)	23.9 (9.22)	416	16.3	–	0.58	–
5	18	5	7	275	29.8 (12.90)	25 (10.82)	556	20.3	1.65	0.94	Surgery for abdomen (3.64)

*Day X refers to the day when severe hypoglycemia occurred

**Average of the body weights on Day 1 and X were used to calculate the value

***The weight of lung abscess, massive pleural effusion, and ascites were subtracted

BG: blood glucose, TGs: triglycerides, BMI: body mass index, eLW/IBW: estimated liver weight/ideal body weight, CRP: C-reactive protein

### Relationship of hypoglycemia with TGs and ALT

**Fig. 6:**
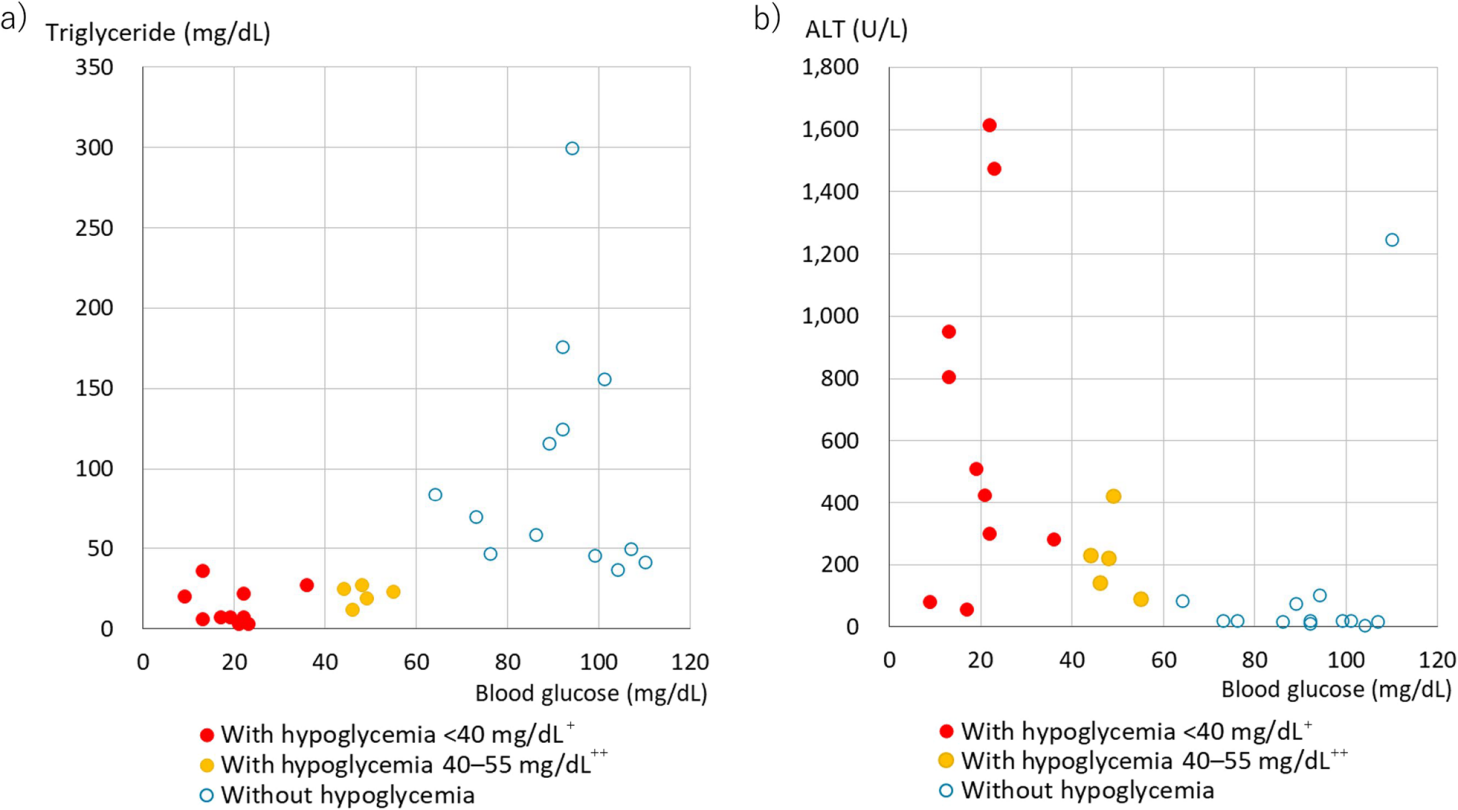
Association of blood glucose with triglycerides and alanine aminotransferase on the day of admission. **a** Correlation between TGs and BG and **b** correlation between ALT and BG. Data from 28 patients who underwent all three tests on the day of admission are presented. ^+^40 mg/dL is equivalent to 2.22 mmol/L. ^++^55 mg/dL is equivalent to 3.05 mmol/L. BG: blood glucose, TGs: triglycerides, ALT: alanine aminotransferase

To confirm the association between hypoglycemia and low TG levels and that between hypoglycemia and liver injury, the values of the three test results for 28 cases in which all three items were measured on the day of admission are shown in Fig. 6. Notably, the 28 cases included 18 and 10 patients admitted to the OGMC and at previous hospitals, respectively. If BG levels were measured more than once per day, the lowest value was used. All 15 patients admitted for hypoglycemia exhibited lower blood TG levels than did those without hypoglycemia (median, 19 mg/dL for those with hypoglycemia and 70 mg/dL for those without hypoglycemia). Among all 28 cases, there was a high correlation between BG and TGs (Spearman’s rank correlation coefficient: 0.76, *p* < 0.001). Moreover, ALT levels were above the normal range in all 15 patients with hypoglycemia, which was more apparent in patients with hypoglycemia lower than 40 mg/dL (2.22 mmol/L) than in those with hypoglycemia with the level of 40–55 mg/dL (2.22–3.05 mmol/L) (median, 466.5 U/L and 223 U/L for those with severe and moderately severe hypoglycemia, respectively). Furthermore, nine of 13 patients without hypoglycemia showed normal ALT values (median, 21 U/L). A strong inverse correlation was also observed between BG and ALT levels in all 28 patients (Spearman’s rank correlation coefficient: -0.59, *p* = 0.002).

### Comparison of groups with and without hypoglycemia

The baseline characteristics of the total participants with or without hypoglycemia (BG < 55 mg/dL [3.05 mmol/L]) are shown in Table 3. Significant differences were observed in the proportion of AN subtypes, BMI at admission and discharge, nadir BMI during the course, and prognosis.

**Table 3:** Baseline characteristics of the study participants with and without hypoglycemia

	With hypoglycemia	Without hypoglycemia	Difference between the two groups
	n = 32	n = 16	*p* values
Sex: male/female	1/31	1/15	0.600
Age	33.5	30.5	0.718
Duration of the disease (years)	7	4.5	0.458
Subtypes of anorexia nervosa Restrictive type/(Binge-eating/purging type)	28/4	9/7	0.015*
The first unit of admission in OGMC (Emergency/Other physical/Psychiatric)	12/2/18	5/1/10	
Duration of admission in previous hospitals (days)	13.5 (n = 18)	20 (n = 7)	0.760
Duration of admission in OGMC (days)	108.5	88	0.450
BMI			
At the time of admission to OGMC	10.16 (n = 29)	11.82	0.010*
The smallest BMI during the first 2 months	9.45	11.20	0.002**
At the time of discharge from OGMC	12.80 (n = 24)	14.37	0.025*
Main reasons for admission			
Hypoglycemia with loss of consciousness	13	0	
Hypokalemia	2	3	
Infection	4	1	
Other complications	7	9	
Severe malnutrition itself	6	3	
Prognoses at the discharge from OGMC [died/hypoglycemic encephalopathy] /well	[6/2]/24	[0/0]/16	0.028*

Hypoglycemia is defined as blood glucose levels < 55 mg/dL (< 3.05 mmol/L). Numerical data are expressed as median

*p < 0.05, **p < 0.01

BMI: body mass index, OGMC: Osaka General Medical Center

Laboratory investigation results of study participants with and without hypoglycemia (BG < 55 mg/dL [< 3.05 mmol/L]) are shown in Table 4. Notably, if data for the initial test were missing, but the item was tested the next day, it was used as the initial result. A comparison of the test results between the two groups demonstrated that, initially, TG, BS, AST, and ALT levels, PT%, and platelet count were significantly lower, whereas, regarding the poorest outcomes within 60 days, almost all parameters, except serum P and K levels, were markedly inferior in the group experiencing hypoglycemia than in the group without hypoglycemia. The levels of albumin and blood cell counts were not markedly disturbed in the initial examination; however, they began to decrease significantly following the initiation of treatment.

**Table 4:** Laboratory investigation results of study participants with and without hypoglycemia

Investigation items		The initial results			The poorest results within 60 days		
		With hypoglycemia	Without hypoglycemia	Difference between the two groups	With hypoglycemia	Without hypoglycemia	Difference between the two groups
(L: lowest, H: highest)	normal range	n = 32	n = 16	*p* values	n = 32	n = 16	*p* values
Albumin (L)	4.1–5.1 g/dL	3.45	3.5 (n = 15)	0.102	1.8	2.5	0.000***
Prealbumin (L)	22–34 mg/dL	8.55 (n = 10)	15.9 (n = 7)	0.625	8.05 (n = 28)	14.8 (n = 12)	0.000***
Triglyceride (L)	30–117 mg/dL	22 (n = 19)	89 (n = 12)	0.000***	20 (n = 30)	42 (n = 15)	0.005**
Glucose (L)	56–108 mg/dL	50 (n = 31)	93 (n = 15)	0.023*	28	70.5	0.000***
Total bilirubin (H)	0.4–1.5 mg/dL	0.9	0.8 (n = 15)	0.522	1.9	0.9	0.004**
AST (H)	13–30 U/L	170	28	0.000***	459	63	0.000***
ALT (H)	7–23 U/L	143	27	0.001**	294.5	56.5	0.000***
Cholinesterase (L)	201–421 U/L	142.5 (n = 20)	169 (n = 14)	0.563	72 (n = 31)	137 (n = 15)	0.000***
Prothrombin time% (L)	86–124.1%	56.3 (n = 24)	82.3 (n = 9)	0.019*	47.3	81.95 (n = 14)	0.000***
Phosphate (L)	2.7–4.6 mmol/L	3.6 (n = 29)	3.65	0.361	2.05	1.9	0.693
Magnesium (L)	1.8–2.4 mmol/L	2.1 (n = 26)	2.15 (n = 14)	0.542	1.55	1.7	0.033*
Potassium (L)	3.6–4.8 mmol/L	3.6	3.5	0.504	2.85	2.95	0.399
CRP (H)	0–0.14 mg/dL	0.04 (n = 31)	0.03 (n = 14)	0.676	5.6	0.545	0.012*
White blood cell (L)	3300–8600 /mm^3^	5750	5950	0.869	2250	3400	0.012*
Hemoglobin (L)	11/6–14.8 g/dL	12.35	11.5	0.196	6.6	8.4 (n = 15)	0.009**
Platelet (L)	158–348 × 1000/mm^3^	140.5	210	0.029*	33	134	0.005**
Total Lymphocyte (L)	1100–2900 /mm^3^	1000 (n = 25)	1100 (n = 12)	0.592	200 (n = 30)	800 (n = 15)	0.000***

Hypoglycemia is defined as blood glucose levels < 55 mg/dL (< 3.05 mmol/L). The median is used for representative values

*p < 0.05, **p < 0.01, ***p < 0.001

AST: aspartate aminotransferase, ALT: alanine aminotransferase, CRP: C-reactive protein

To clarify the decline in albumin levels and blood cell systems after the start of treatment, Table 5 shows the number of cases in which blood products were administered during the treatment and the day of the first administration (if there was a previous hospitalization, the number of days from the previous physician’s admission was used). These cases were more common in the hypoglycemia group, and the median date of first administration was 10–20 days after admission.

**Table 5:** Transfusion of blood products: number of cases and the day of initial administration

	With hypoglycemia n = 32	Without hypoglycemia n = 16	Difference between the two groups
Albumin transfusion	6	1	*p* = 0.247
Day of initial transfusion (median; range)	10.5 (2–17)	14	
Red blood cell transfusion	16	3	*p* = 0.036*
Day of initial transfusion (median; range)	20 (1–42)	10 (1–22)	
Platelet transfusion	8	0	*p* = 0.028*
Day of initial transfusion (median; range)	11.5 (3–80)		

Hypoglycemia is defined as < 55 mg/dL (< 3.05 mmol/L)

*p < 0.05

## Discussion

### Implications of reduced liver volume

The hypothesized implication and importance of liver volume reduction in the pathophysiology of severe malnutrition were examined and confirmed from multiple perspectives. To the best of our knowledge, this is the first study to examine liver volume in severe malnutrition. Focusing on the temporal relationship between hypoglycemia and the date of CT, we found that reduced liver volume was almost always associated with severe hypoglycemia (Figs. 3, 4, 5). In addition, hypoglycemia on admission was always accompanied by elevated ALT (Fig. 6b). Furthermore, the most unfavorable blood test outcomes over a 60-day period for all liver function-related parameters (Alb, AST, ALT, ChE, PT% [*p* < 0.001] and total bilirubin and platelet counts [*p* < 0.01]) were significantly poorer in the hypoglycemic group than in the non-hypoglycemic group (Table 4). These findings indicate that reduced liver volume is not a simple issue of size but is associated with serious liver dysfunction, including hypoglycemia.

In the five patients who showed severe hypoglycemia 13–18 days after admission to the OGMC, the amount of nutrients administered during this period was very low, and in four patients, the liver volume was markedly reduced during this period (Fig. 5a and Table 2). In three of these patients, as well as in 15 patients admitted with hypoglycemia, markedly low levels of TGs were concurrently observed in the blood (Table 2 and Fig. 6a). This implies that severe hypoglycemia manifests subsequent to the body's depletion of energy sources, encompassing TGs in the bloodstream, along with a shortage of external nutrients, and suggests that the precipitous reduction in liver volume immediately preceding hypoglycemia may indicate that the liver is utilized as an energy source. Figure 4, which illustrates the correlation between liver volume and BMI, reveals that the steep inclination of the regression line for patients with AN could signify liver consumption at a BMI lower than 13 kg/m^2^.

Liver volume from the control group with a median BMI of 20.85 kg/m^2^ suggested a normal liver eLW/IBW of 2.0%–2.5% (median: 2.22%) (Fig. 3). The threshold value of 1.08%, at which hypoglycemia occurred at an increased rate (Fig. 3), was approximately half the size of the native liver. Importantly, glycogen depletion in the liver can reduce its volume. However, the amount of glycogen that can be stored in the liver is approximately 100 g [22]; hence, all the volume reduction of the liver observed in this study could not be explained.

Notably, starvation-induced liver autophagy inevitably occurs in severely malnourished situations [16]. Rautou et al., who documented liver autophagy, included 12 patients with a PT% less than 50% and a median BMI of 11.9 kg/m^2^ [16]. Among them, four patients in whom autophagy findings were confirmed via electron microscopy exhibited a median BMI of 10.7 kg/m^2^ and a median PT% of 35%. In contrast, in this study, the 32 patients in the hypoglycemia group presented a median BMI upon admission of 10.16 kg/m^2^ and a median PT% of 47.3%, indicating that at least half of the patients with hypoglycemia had a PT% below 50%, mirroring the condition observed in the study by Rautou et al.

### Hypoglycemia as an indicator of complications and poor prognosis

The poorest values in the first 60 days of treatment in the group with hypoglycemia were significantly worse than those in the group without hypoglycemia in most of the blood investigation items; prealbumin and total lymphocyte count exhibited significant associations (*p* < 0.001), as did TGs and hemoglobin (*p* < 0.01), along with serum magnesium and white blood cell count (*p* < 0.05). Furthermore, all the liver function-related parameters previously discussed were also significantly associated. However, the lowest serum P and K levels did not differ significantly between the two groups (Table 4). Moreover, transfusion of blood products was performed more frequently for patients with hypoglycemia than for those without hypoglycemia (Table 5). Delayed decrease of albumin, hemoglobin, and platelet is considered to reflect their disturbed production due to the hypoglycemic crisis, as these items are constantly replaced, and their blood levels are maintained by continuous production. These findings indicate that a range of physical complications encountered during re-nutrition is more closely associated with the pathology of malnutrition itself represented by hypoglycemia than with the narrowly defined refeeding syndrome, the primary pathogenesis of which is hypophosphatemia. In some other instances, thrombocytopenia in patients with AN is reportedly caused by decreased thrombopoietin production in the liver due to severe liver dysfunction [23]. Moreover, takotsubo cardiomyopathy and cardiac arrest may be associated with hypoglycemia, as well as low blood TGs, concomitant with elevated catecholamine levels in the blood [24, 25].

Furthermore, liver failure and severe hypoglycemia are associated with poor prognosis. Rich et al. [26], drawing from their own experience and previous studies, reported that seven of 10 patients with hypoglycemia died. In our study, all six deaths during hospitalization occurred in the hypoglycemia group, and hypoglycemic encephalopathy occurred in two other cases (Table 1). The post-discharge prognosis based on the information obtained from family members, related institutions, and details upon readmission to OGMC or other hospitals revealed that eight additional deaths and two additional cases of hypoglycemic encephalopathy occurred within 2 years of discharge; all 10 of these cases occurred in the hypoglycemia group. Severe hypoglycemia, accompanied by hepatic volume reduction and associated complications, constitutes the pathophysiology of the terminal stage of severe malnutrition, which is a major cause of death in AN. Refeeding syndrome is a well-defined concept that can lead to life-threatening complications; however, severe malnutrition itself is considered to have another pathophysiology closely related to poor prognosis.

### Risk of undernutrition

A very small quantity of nutrition should not be sustained for an extended duration, as it may instigate and advance liver consumption. Importantly, during the initial years of the research period, we started minimal doses of nutrition and subsequently escalated them too judiciously. The 2006 edition of the NICE guideline recommends: “Start feeding 0.0418 MJ (10 kcal)/kg/day. Slowly increase feeding over 4–7 days. If the patient is severely malnourished (for example, BMI < 14 kg/m^2^), or if the intake is negligible for > 2 weeks, start feeding at maximum of 0.0209 MJ (5 kcal)/kg/day.” [3] Nevertheless, the guideline does not specify the duration for which low-calorie nutrition can be sustained. Moreover, it was posited that the feeding rate should be slowed down if refeeding syndrome is detected and that essential electrolytes should be administered [3]. In five patients who exhibited hypoglycemia on days 13–18 post-admission, an extremely low nutrient intake, averaging 252–556 kcal/day (approximately 10–20 kcal/kg/day), was maintained. One patient among them experienced severe hypoglycemia shortly after a reduction in nutritional intake, consequent to a gradual elevation in liver enzymes.

Rigaud et al. [27] measured the resting metabolic rate of 41 patients with AN with a BMI of 12.1 ± 1.5 kg/m^2^ at admission and reported it to be 845 ± 51 kcal/day. Additionally, Gentile et al. [28] measured the resting metabolic rate of 33 patients with AN with a BMI of 11.2 ± 0.7 kg/m^2^ and reported it to be 776 ± 145 kcal/day. Although not an exact analogy, simply dividing these values by the patients’ average admission weight yields values of 24.9 ± 1.5 kcal/kg/day and 26.7 ± 5.0 kcal/kg/day based on the studies of Rigaud et al. [27] and Gentile et al. [28], respectively. Per Scalfi et al. [29], who measured the basal metabolic rate of 120 female patients with AN in their teens and 20s, the resting metabolic rate of patients weighing 25–35 kg can be estimated to be 23–24 kcal/kg/day on average. Therefore, 25 kcal/kg/day might be an approximate basal metabolic rate in severely malnourished patients. Notably, energy consumption can increase when inflammation, trauma, or other physical illnesses occur.

Recent recommendations advocate initiating refeeding with a high caloric intake (1400 kcal/day or more) and escalating it rapidly, as this approach did not increase the risk of complications; rather, it reduced the hospital stays [30]. However, such nutritional therapies are designed for patients with a BMI of 15–17 kg/m^2^ [31–34] whose pathophysiology differs from that of severe malnutrition. Moreover, Garber et al. [35], in a systematic review, suggested that there is insufficient evidence to assert that a high-calorie intake is superior in cases of severe malnutrition. Patients suffering from severe malnutrition with smaller livers should be more susceptible to nutritional load than those with normal-sized livers. In this study, two cases treated by other physicians indicated that an overly rapid increase in nutrients can cause fatty liver and hepatomegaly, alongside other complications.

We did not measure the resting metabolic rate; instead, we assumed that the minimum nutritional requirement for day-to-day survival was approximately 700–800 kcal/day. Moreover, we posited that an extremely low nutritional intake during the initial days could exacerbate malnutrition. Conversely, refeeding syndrome, caused by excessive carbohydrate intake, must be avoided. Thus, we commenced with an intake of approximately 500 kcal/day, progressively increasing it to 800 kcal/day in a week. Importantly, the patient's physical condition stabilized when the intake was augmented to 1200 kcal after > 1 additional week. An increase in nutrients, including fatty acids and proteins (amino acids), along with phosphorus and potassium supplementation, could prevent deaths from undernutrition. Notably, this refeeding schedule for patients with severe malnutrition was informed by our experience and personal communications with experts in this condition. The verification of the re-nutrition protocol for such patients is beyond the scope of this study and warrants future investigation.

### Liver pathophysiology and treatment

The liver, having lost volume, loses its capacity to regulate BG by rapidly converting ingested carbohydrates into glycogen for storage and subsequently releasing them into the bloodstream as needed. Hence, dosing regimens that substitute for the BG-regulating ability of the liver are of vital importance. Continuous 24-h dosing is the best, and in the case of oral intake, small, frequent doses are preferable. We encountered a case with hypoglycemia where liver enzyme levels rose after three oral doses per day but declined after six smaller doses per day. Notably, short-term, high-dose carbohydrate administration significantly burdens the liver, leading to elevated liver enzyme levels, pronounced hyperglycemia and insulin secretion, and a risk of hypoglycemia and refeeding syndrome due to excess insulin.

Furthermore, liver failure with reduced liver volume cannot tolerate fasting, and severe hypoglycemia can occur quickly. Thus, consuming midnight snacks for at least the first 2 weeks may be beneficial when sustained nutrition is lacking. One of the five patients with severe hypoglycemia after hospitalization refused to eat after breakfast, resulting in a deep coma the next morning due to severe hypoglycemia.

Carbohydrates not only burden the liver but also diminish blood P levels, consequently elevating the risk of refeeding syndrome [36, 37]. Lipids are regarded as the primary energy source during starvation and, notably, do not reduce blood P levels through insulin secretion. Additionally, animal studies indicate that liver autophagy elevates blood amino acid concentrations, and gluconeogenesis is initiated from amino acids [38]. Therefore, even amid hepatic contraction and liver failure, the preservation of BG levels using lipids and proteins (amino acids) can be sustained. Several cases in this study supported the benefit of lipid and amino acids to stabilize BG levels (data not shown). Although hypoglycemic episodes necessitate immediate glucose administration, a reassessment of the nutrition protocol is imperative to avert hypoglycemia recurrence.

### Limitations

Our study had several limitations that need to be considered. First, it was a single-center study. The pathophysiology of malnutrition may have been more prominent due to the administration of very low nutritional doses at our institution, especially in the early study period. Second, due to the retrospective observational nature of the study, missing values were unavoidable. Third, this study covers a span of 14 years, during which the treatment members and methods have evolved. However, two of the authors have been actively involved with OGMC for nearly the entirety of the study period. Fourth, the evaluation of liver volume was performed preliminarily. The reference values for liver volume in healthy participants and other factors affecting liver volume warrant investigation in future studies. Finally, liver biopsy was not performed in this study; therefore, microscopical evidence of liver autophagy was not confirmed in our cohort.

## Conclusions

A reduction in liver volume was associated with hypoglycemia, low serum TG levels, and liver dysfunction in patients with severe malnutrition, occurring in response to a deficiency in external nutrients followed by the exhaustion of the body's energy sources. It was postulated that this decrease in liver volume resulted from liver consumption, i.e., liver autophagy, which represents a final mechanism to procure energy essential for survival; however, infringing upon vital organs may precipitate imminent death. Importantly, the pathophysiology of end-stage malnutrition is closely linked with complications encountered during re-nutrition. In managing these patients, it is crucial to avoid extremely low nutrient levels, such as 5–10 kcal/kg/day.

## Data Availability

The dataset supporting the conclusions of this article is included within the article.

## Abbreviations

ALT: Alanine aminotransferase
AN: Anorexia nervosa
AST: Aspartate aminotransferase
BG: Blood glucose
BMI: Body mass index
ChE: Cholinesterase
CRP: C-reactive protein
CT: Computed tomography
eLW/IBW: Estimated liver weight/ideal body weight
K: Potassium
Mg: Magnesium
NICE: National Institute for Health and Care Excellence
OGMC: Osaka General Medical Center
P: Phosphorus
PT%: Prothrombin time %
TGs: Triglycerides
